# Establishing the feasibility of assessing the mental health of children displaced by the Syrian conflict

**DOI:** 10.1017/gmh.2015.3

**Published:** 2015-06-19

**Authors:** K. Cartwright, A. El-Khani, A. Subryan, R. Calam

**Affiliations:** School of Psychological Sciences, The University of Manchester, Zochonis Building, Brunswick Street, Manchester, M13 9PT, UK

**Keywords:** Children, conflict, feasibility, mental health, Syria

## Abstract

**Background.:**

In the humanitarian crisis context of conflict zones, collecting data is essential for identifying and addressing the mental health needs of refugee children to avoid mass suffering. This study tested the feasibility of recruiting refugees caring for children and using established and brief parent-report questionnaires in a challenging context to collect mental health data on refugee children displaced by Syria's conflict.

**Methods.:**

Caregivers of 4–10-year olds attending primary schools run by non-governmental organisation (NGO) Generation Freedom in and near refugee camps on the Syrian–Turkish border were invited to complete the Pediatric Emotional Distress Scale (PEDS) and Strengths and Difficulties Questionnaire (SDQ).

**Results.:**

It was possible to reach 144 adult refugees caring for children with research participation information and use informed consent procedures. A total of 106 caregivers completed the questionnaires yielding a good return rate (74%). Eighty-two (77.4%) caregivers had complete data on the PEDS and 61 (57.5%) on the SDQ. Almost half (49%) of the children met the clinical cut-off for being anxious/withdrawn and 62% for being fearful rated using the PEDS and 45% for SDQ rated emotional symptoms. More than a third had clinical levels of behavioural problems on both scales.

**Conclusions.:**

It proved feasible to collect child mental health data in challenging conditions in the context of the Syrian crisis with support from a local NGO providing humanitarian assistance. The PEDS performed better than the SDQ in this context. High levels of emotional distress and behavioural problems in children reiterate the urgent need for evidence-based psychosocial support.

## Introduction

It is 4 years since civil unrest and fighting broke out in Syria in the Middle East. The conflict has developed into a humanitarian and public health catastrophe, resulting in extraordinary levels of violence, human rights violations and millions being uprooted from their homes and forced into severe deprivation where resources are limited and living conditions poor. Over 12 million people need humanitarian assistance inside Syria (7.6 million are internally displaced) and nearly four million have been externally displaced to neighbouring countries Turkey, Jordan and Lebanon (UNHCR, 2015). Half of those affected are children. The risk of children becoming the ‘lost generation’ is recognised as a serious concern (UNICEF, 2013).

It is well documented that children exposed to war and political violence are more vulnerable to mental health problems as a consequence, in particular, posttraumatic stress disorder (PTSD) and depression (Fazel & Stein, 2002; Quota *et al.* 2003; Lustig *et al.* 2004; Fazel *et al.* 2005; Attanayake *et al.* 2009; Bronstein & Montgomery, 2011; Dimitry, 2011) with refugee children living in camps as opposed to urban or rural areas more likely to suffer (Khamis, 2005). Children are also at a higher risk of developing behavioural problems, especially aggression, as a result of the traumatic events that they have witnessed or been a victim to (Quota *et al.* 2008). The onset of these problems in childhood has a long-term impact on the child, their family, friends, society and future generations. Building resilience and optimising child and adolescent mental health, especially in humanitarian settings, has high priority on international research agendas for prevention and treatment (Mollica *et al.* 2004; Tol *et al.* 2011b; Tol & van Ommeren, 2012), as poor mental health accounts for a significant proportion of disease burden and disability among children and adolescents worldwide (Patel *et al.* 2007, 2008; UNICEF, 2005).

In conflict zones, recruiting participants into research studies and collecting reliable and valid data are complex, challenging and can be near impossible in some settings. The specific nature, intensity and length of particular conflicts, the challenges this presents and impact it has on infrastructure, routine and normal lifestyle together with different cultures across war-affected countries makes it difficult to transfer data collection approaches from one conflict to another. A recent systematic review undertaken by the United Nations High Commission for Refugees found that there are no peer-reviewed published studies on the impact of the conflict in Syria on the mental health of refugees and those internally displaced (Quosh *et al.* 2013). Thirteen studies, five quantitative and eight qualitative, were identified from the grey literature which included a diversity of document types produced by humanitarian and non-governmental agencies and organisations, academics and governments, ‘where publishing is not the primary activity of the producing body’. Less than half of the studies that met the inclusion criteria for the review focused specifically on children. Insecurity was reported as causing difficulties in conducting assessments inside Syria. Furthermore, contexts of conflict can pose serious risks to the safety of researchers and participants. It is critical that feasible and ethical recruitment and data collection approaches that can be used safely in the Syrian conflict are identified rapidly as part of the essential process of reliably identifying levels of mental health difficulties among children, so that screening and pre- and post-intervention measures can be administered to identify children's psychological needs and evaluate the effectiveness of interventions used to address them. The primary aim of this study therefore was to test the feasibility of recruiting refugee caregivers of children affected by the Syrian conflict living in and near refugee camps on the Syrian–Turkish border into a questionnaire-based study and of collecting reliable data from them.

The second aim of the study was to examine the extent to which complete data could be obtained from refugee caregivers under field conditions such as those experienced in and near refugee camps, and to identify the level of help parents needed to complete measures. Extreme levels of stress, poor physical and/or mental health, literacy difficulties, poor living conditions and insecurity may contribute to refugees finding it difficult to complete questionnaires. Collecting data in contexts of conflict require psychometrically sound, culturally sensitive and developmentally appropriate screening instruments that are inexpensive, can be quickly and accurately measured, are non-burdensome and require limited training to administer. Two measures which fit these criteria were chosen for the current study.

The Pediatric Emotional Distress Scale (PEDS; Saylor *et al.* 1999), which was designed specifically to measure trauma-related symptoms in children as young as 2 years of age, can be completed in 5–8 minutes and has acceptable psychometric properties as found in independent, geographically and developmentally diverse samples of children exposed or unexposed to traumatic events, including natural disasters and sexual abuse (Stokes *et al.* 1995; Saylor *et al.* 1999). We are not aware of any published data on the PEDS used in conflict.

The Strengths and Difficulties Questionnaire (SDQ; Goodman, 1997) is an inexpensive, brief, user-friendly and effective screening measure of child behavioural, emotional and social problems. It is well established, translated into over 60 languages with normative data available in 10 high-income countries and has been validated in many contexts internationally allowing comparisons to be made between different populations. It is the only widely used mental health scale to have been validated in Arabic. While not trauma specific, it has been used with mixed success in studies to assess the impact of conflict-related trauma on behaviour in children resettled in high-income countries (Papageorgiou *et al.* 2000; Fazel & Stein, 2003; Derulyn & Broekaert, 2007; Nielsen *et al.* 2008; Vaage *et al.* 2009; Durà-Vilà *et al.* 2012) and during the flight phase in displaced children exposed to war in the Middle East ( Thabet *et al.* 2000, 2006) and Afghanistan (Panter-Brick *et al.* 2009). It has also been used to measure the effect of psychological intervention on post-traumatic stress in Palestinian children in randomized controlled trials (Quota *et al.* 2012; Barron *et al.* 2013).

The third aim of the study was to gain insight into emotional and behavioural difficulties among refugee children living in and near refugee camps on the Syrian–Turkish border. The Bahçeşehir Study, a cross-sectional survey conducted in Islahiye Refugee Camp, Turkey (housing over 4000 children) found that in a sample of 185 girls and 103 boys aged 9–18 years 45% were experiencing parent-reported symptoms of PTSD, 60% depression, 22% aggression and 65% psychosomatic symptoms that seriously reduced their level of functioning (Özer *et al.* 2013). Seventy four per cent of these children had experienced the death of somebody they cared about and 50% had been exposed to six or more traumatic events. Mobayed (2014) (cited in Abou-Saleh & Mobayed, 2013) found that in two refugee camps the prevalence of PTSD among 129 Syrian children aged 10–16 years was 41%–76%; these rates were higher than those for adults (36%–62%).

This study therefore aims to contribute to programmes of research in the Syrian conflict identifying refugee children's mental health needs accurately and reliably in order to evaluate interventions used to address them.

## Materials and methods

### Participants

Participants were adults caring for children aged 4–10 years and who had been forced to flee their homes due to armed conflict in Syria. Table 1 shows their characteristics. They were recruited from primary schools run by Generation Freedom in two different refugee camps, Qah Refugee Camp and Bab-Al-Salam Refugee Camp, in two locations in the northern part of Syria, and near a refugee camp in Reyhanli in southern Turkey away from immediate armed conflict. Qah Refugee Camp was established in 2012 and hosts more than 20 000 refugees. The continual movement of families on a day-to-day basis means that these figures have likely changed since this was written.

**Table 1.** Sample characteristics

Demographic characteristic	*N*	%
*Child age*^a^		
Mean (s.d.)	7.46 (1.67)	–
Range	4–10	–
*Child gender*		
Male	53	50.0
Female	53	50.0
*Primary caregiver relationship to child*		
Mother (biological or adoptive)	79	74.5
Father (biological or adoptive)	15	14.2
Step/foster-mother^b^	6	5.7
No response	6	5.7
*Primary caregiver's marital status*		
Married	86	81.1
Separated/divorced	3	2.8
Single	3	2.8
Widowed	12	11.3
No response	2	1.9
*Family structure*		
Original family (both biological or adoptive parents present)	86	81.1
Step family (one being a biological parent, one being a step parent)	1	0.9
Foster family (one being a biological parent, one being an aunt)^c^	5	4.7
Single parent family^d^	14	13.3
*Primary caregiver education*		
Primary school or less	18	17.0
Some or completed high school	33	31.1
Trade/technical college/university degree or higher	52	49.1
No response	3	2.8
*Partner education*		
Primary school or less	12	11.3
Some or completed high school	30	28.3
Trade/technical college/university degree or higher	59	55.7
No response	5	4.7
*Primary caregiver occupational status (previous/current)*		
Full-time	28/17	26.4/16.0
Part-time	17/14	16.0/13.2
Not working, looking for a job	4/33	3.8/31.1
Home-based paid work^e^	42/27	39.6/25.5
Not working^f^	12/14	11.3/13.2
No response	3/1	2.8/0.9
*Partner occupational status (previous/current)*		
Full-time	76/29	71.7/27.4
Part-time	7/12	6.6/11.3
Not working, looking for a job	8/27	7.5/25.5
Home-based paid work	8/7	7.5/6.6
Not working	2/12	1.9/11.3
War (current only)	8	7.5
No response or not applicable	5/11	4.7/10.4
*Length of time displaced*		
Less than 3 months	19	17.9
3–6 months	24	22.6
6–12 months	43	40.6
More than 12 months	20	18.9
*Current housing situation*		
Refugee camp	37	34.9
House/apartment	62	58.5
Refugee building	5	4.8
Other	1	0.9
No response	1	0.9
*Number of people in household*		
1–5	52	49.1
6–12	46	43.4
100 or more	5	4.7
No response	3	2.8
*Income*		
Yes	49	46.2
No	53	50.0
No response	4	3.8

a Missing value for one participant.

b 1 step-mother, 5 aunts.

c 1 aunt was a widow.

d 1 spouse absent, 2 separated/divorced, 11 widowed.

e Child care, sewing, internet/phone-based work etc.

f includes stay-at-home parents, being retired.

The primary caregiver was the adult who had primary responsibility for caring the children and was also the respondent who completed the questionnaire.

Generation Freedom is a sub-organisation of Watan who specialise in educating children aged between 7 and 17. Watan is a Syrian non-governmental organisation (NGO) working internationally and locally in Turkey, Syria, Jordan and Lebanon to provide humanitarian assistance to people affected by the Syrian conflict. The locations for the current study were chosen for practical and security reasons. Generation Freedom has extensive knowledge and experience of working in these locations having provided humanitarian assistance here since the conflict started. This allowed the researcher to safely cross the border and access the camps and schools.

### Measures

#### Family background questionnaire

An abbreviated, adapted version of the Family Background Questionnaire (FBQ) (Sanders *et al.* 2000) was used to obtain demographic information, both general (e.g. child age, gender and relationship to caregiver) and context-specific (e.g. length of time displaced and current living situation).

#### PEDS

The PEDS (Saylor *et al.* 1999), completed by parents, comprises 17 general behaviour items rated on a Likert scale from 1 (‘Almost never’) to 4 (‘Very often’) which contribute to three subscales: Anxious/Withdrawn (six items) (e.g. ‘Seems sad and withdrawn’), Fearful (five items) (e.g. ‘Refuses to sleep alone’) and Acting out (six items) (e.g. ‘Has temper tantrums’). There are an additional four trauma-specific items. Total scores, based on the first 17 items, range from 21 to 84. Total PEDS and Acting Out scores have been found to correlate with the Eyberg Child Behaviour Inventory (*r* = 0.62, *p* < 0.001 and *r* = 0.86, *p* < 0.001, respectively) (Frederick, 1985a). Sensitivity to change and convergent and discriminant validity in clinical samples (based on a cut-offs determined through use of the DSM-IV-TR) have been found (Saylor *et al.* 1999). Cronbach's alphas in the current study were 0.72 for Anxious/Withdrawn, 0.75 for Fearful, 0.72 for Acting Out and 0.83 for the total score.

#### Strengths and difficulties questionnaire

The parent version of the SDQ (Goodman, 1997) for 4–16-year olds screens for behavioural, emotional and social problems based on ICD-10 and DSM-IV criteria using 25 items rated on a 3-point Likert scale ranging from 0 (‘Not True’) to 2 (‘Certainly True’). Single informant ratings have shown satisfactory reliability and validity in high-income countries (Goodman, 2001) and across culturally diverse settings including Bangladesh (Goodman *et al.* 2000; Mullick & Goodman, 2001), Pakistan (Samad *et al.* 2005; Syed *et al.* 2007) and Yemen (Alyahri & Goodman, 2006) where the Arabic version of the questionnaire demonstrated adequate discrimination between community and clinic 5–10-year-old children. Internal consistency in the current study was *α* = 0.57 for Emotional Symptoms, *α* = 0.59 for Conduct Problems, *α* = 0.60 for Hyperactivity, *α* = 0.38 for Peer Problems, *α* = 0.82 for Prosocial Behaviour and *α* = 0.66 for Total Difficulties.

### Procedure

Ethics approval for the study was obtained from The University of Manchester. The authors assert that all procedures contributing to this work comply with the ethical standards of the relevant national and institutional committees on human experimentation and with the Helsinki Declaration of 1975, as revised in 2008. This included very detailed risk assessments. Participant information sheets, consent forms and the PEDS were translated into Arabic with back-translation and printed in the UK before being taken by the researcher (A–E) to Generation Freedom run makeshift primary schools in or near refugee tented camps on the Syria–Turkey border. Translation and back-translation of the PEDS were carried out by two translators at a professional translation service. One translator conducted the translation and the other the back-translation. Discrepancies were discussed and resolved between the translators. Generation Freedom staff supported the researcher with border crossings, access to the refugee camps and schools during data collection and maintained the researcher's safety. Data were collected over a period of 23 days between March and April 2013 with 1 week spent in each location. Following permission to approach parents from the Director of Generation Freedom, parents were approached by the researcher on a one-to-one or group basis. The school in the Qah Refugee Camp was located in a large building and in five purpose-built tents in the Bab As-Salam Camp. In Reyhanli, data were collected in a building housing 700 refugees across the road from a school and near to a refugee camp. Primary caregivers of children were given a participant information sheet and the opportunity to ask questions. Those who wished to take part were given a consent form and the questionnaires and either completed both immediately at the school or took them away and returned them the following day.

### Data analysis

Data were analysed using IBM SPSS Statistics for Windows, Version 22 (IBM Corp, 2013). First, descriptive statistics for complete and missing data on the FBQ, PEDS and SDQ were obtained including the proportion of: (1) participants with complete and missing values on at least one item of the questionnaires, (2) items with complete and missing values on each questionnaire; (3) complete and missing values across all items of the questionnaires. Descriptives of completeness of data were obtained for participants who were not helped by the researcher to complete the questionnaires *v.* those who were helped by the researcher. Second, internal consistency of the PEDS and SDQ was established using Cronbach's alpha coefficients for the scales as a whole as well as the individual subscales. Items on the PEDS and SDQ with less than 10% incomplete data were replaced with the mean for that item before Cronbach's alpha coefficients were calculated. One participant was excluded from the reliability analysis for the SDQ due to 15 incomplete items. The reliability analysis for the SDQ Total Difficulties and Hyperactivity scales excluded item 25 due to the amount of incomplete data for this item (27.4%). Finally, means, standard deviations and range of scores on each of the subscales of the PEDS and SDQ, their total scores and the proportion of children with scores at or above clinical cut-offs were calculated. The frequencies of responses to each of the four trauma-specific items of the PEDS were also calculated.

## Results

### Feasibility of recruitment and distributing and receiving back questionnaires

The researcher was able to distribute written participant information about the study and provide a verbal explanation of the study to caregivers of children at primary schools in and near refugee camps without causing apparent additional distress to parents. It was possible to obtain written informed consent for participation. Questionnaires were distributed to 144 parents and completed and returned by 106 parents (response rate 74%).

### Extent of completion of measures

The researcher helped 65 parents to complete the questionnaires and the remaining 41 parents completed the questionnaires without the researcher's assistance.

#### FBQ

For the FBQ, 83 (78.3%) caregivers had completed all items of the questionnaire. Four (23.5%) of the 17 items of the questionnaire had complete data from every participant. 41(2.3%) values were missing across all items of the questionnaire. Items with the most omitted responses related to primary caregiver relationship to child, partner education, partner previous and current occupational status and questions responded to with ‘other’ which required further elaboration.

#### PEDS

For the PEDS, 82 (77.4%) parents had complete data; 24 (22.6%) had omitted a response to at least one item (Table 2). Data were complete for 8 (38.1%) of the 21 items. Twenty six values (1.2%) were missing across the 21 items of the scale across all participants. Responses to item 13 ‘Gets frustrated too easily’ were the most frequently omitted with six parents not completing this item. This was followed by item 11 ‘Seems hyperactive’ (missing *n* = 4). These items are both included in the Acting Out scale. Twelve parents did not respond to at least one of the items on this scale, eight did not for the Anxious/Withdrawn scale and five for the Fearful scale.

**Table 2.** Completeness of data on the paediatric emotional distress scale

Item	Scale	Complete data whole sample (*n* = 106)		Complete data completed without researcher (*n* = 41)		Complete data completed with researcher (*n* = 65)	
		*N*	%	*N*	%	*N*	%
1. Acts whiny	Acting out	106	100.0	41	100.0	65	100.0
2. Wants things right away	Acting out	105	99.1	41	100.0	64	98.5
3. Refuses to sleep alone	Fearful	105	99.1	40	97.6	65	100.0
4. Has trouble going to bed/falling asleep	Fearful	103	97.2	39	95.1	64	98.5
5. Has bad dreams	Fearful	106	100.0	41	100.0	65	100.0
6. Seems fearful without good reason	Fearful	105	99.1	40	97.6	65	100.0
7. Seems worried	Anxious/withdrawn	105	99.1	40	97.6	65	100.0
8. Cries without good reason	Anxious/withdrawn	104	98.1	40	97.6	64	98.5
9. Seems sad and withdrawn	Anxious/withdrawn	104	98.1	41	100.0	63	96.9
10. Clings to adults/does not want to be alone	Fearful	106	100.0	41	100.0	65	100.0
11. Seems ‘hyperactive’	Acting out	102	96.2	41	100.0	61	93.8
12. Has temper tantrums	Acting out	106	100.0	41	100.0	65	100.0
13. Gets frustrated too easily	Acting out	100	94.3	38	92.7	62	95.4
14. Complains about aches and pains	Anxious/withdrawn	105	99.1	40	97.6	65	100.0
15. Acts younger than used to for age (i.e. bed-wetting, baby talk, thumb sucking)	Anxious/withdrawn	104	98.1	41	100.0	62	95.4
16. Seems to be easily startled	Anxious/withdrawn	106	100.0	41	100.0	65	100.0
17. Acts aggressive	Acting out	105	99.1	40	97.6	65	100.0
18. Creates games, stories or pictures about …	–	106	100.0	41	100.0	65	100.0
19. Brings up … in conversation	–	106	100.0	41	100.0	65	100.0
20. Avoids talking about … even when asked	–	105	99.1	40	97.6	65	100.0
21. Seems fearful of things that are reminders of …	–	106	100.0	41	100.0	65	100.0

Note. Items 1–17 are included in scale calculations.

Incomplete data on PEDS individual items led to there being 20 participants with incomplete total scores for the Total score, nine for the Anxious/Withdrawn scale and Acting Out scales and three for the Fearful scale.

Data were more complete for participants who completed the PEDS without the help of the researcher than those who were helped by the researcher. Of the 41 parents who completed the PEDS without the help of the researcher, 31 (75.6%) had complete data; ten (24.4%) had missing data including eight with one omitted item and two with two omitted items. Of the 65 parents who were helped by the researcher, 51 (78.5%) had complete data; 14 (21.5%) had missing data on one item.

#### SDQ

For the SDQ, 61 (57.5%) caregivers had complete data; 45 (42.5%) did not respond to at least one question (Table 3). Four items (16%) out of the 25 items were complete across all participants. There were 77 (2.9%) missing values in total across the 25 items of the questionnaire. Responses to item 25 ‘Sees tasks through to the end, good attention span’ were most frequently omitted with 29 parents not completing this item. This was followed by item 3 ‘Often complains of headaches, stomach-aches or sickness’ (missing *n* = 6), item 20 ‘Often volunteers to help others (parents, teachers, other children)’ (missing *n* = 5), item 6 ‘Rather solitary, tends to play alone’, item 12 ‘Often fights with other children or bullies them’ and item 13 ‘Often unhappy, down-hearted or tearful’ (missing *n* = 4 for all three items). Items from the Hyperactivity scale, which item 25 is included in, were the least complete; 33 parents did not respond to one or more of the five items that make up this scale. Items from the Emotional Symptoms scale had the second most incomplete data with 16 respondents omitting responses to one or more of the five items, followed by Peer Problems (*n* = 11), Conduct Problems (*n* = 10) and Prosocial Behavior (*n* = 7).

**Table 3.** Completeness of data on the strengths and difficulties questionnaire

Item	Scale	Complete data whole sample (*n* = 106)		Complete data completed without researcher (*n* = 41)		Completed data completed with researcher (*n* = 65)	
		*N*	%	*N*	%	*N*	%
1. Considerate of other people's feelings	Prosocial	106	100.0	41	100.0	65	100.0
2. Restless, overactive, cannot stay still for long	Hyperactivity	106	100.0	41	100.0	65	100.0
3. Often complains of headaches, stomach-aches or sickness	Emotional symptoms	100	94.3	36	87.8	64	98.5
4. Shares readily with other children (treats, toys, pencils, etc.)	Prosocial	105	99.1	41	100.0	64	98.5
5. Often has temper tantrums or hot temper’	Conduct problems	103	97.2	39	95.1	65	100.0
6. Rather solitary, tends to play alone	Peer problems	102	96.2	38	92.7	64	98.5
7. Generally obedient, usually does what adults request	Conduct problems	105	99.1	40	97.6	65	100.0
8. Many worries, often seems worried	Emotional symptoms	103	97.2	39	95.1	65	100.0
9. Helpful if someone is hurt, upset or feeling ill	Prosocial	106	100.0	41	100.0	65	100.0
10. Constantly fidgeting or squirming	Hyperactivity	104	98.1	40	97.6	64	98.5
11. Has at least one good friend	Peer problems	104	98.1	40	97.6	64	98.5
12. Often fights with other children or bullies them	Conduct problems	102	96.2	40	97.6	62	95.4
13. Often unhappy, down-hearted or tearful	Emotional symptoms	102	96.2	39	95.1	63	96.9
14. Generally liked by other children	Peer problems	105	99.1	40	97.6	65	100.0
15. Easily distracted, concentration wanders	Hyperactivity	106	100.0	41	100.0	65	100.0
16. Nervous or clingy in new situations, easily loses confidence	Emotional symptoms	104	98.1	39	95.1	65	100.0
17. Kind to younger children	Prosocial	105	99.1	40	97.6	65	100.0
18. Often lies or cheats	Conduct problems	104	98.1	39	95.1	65	100.0
19. Picked on or bullied by other children	Peer problems	104	98.1	39	95.1	65	100.0
20. Often volunteers to help others (parents, teachers, other children)	Prosocial	101	95.3	37	90.2	64	98.5
21. Thinks things out before acting	Hyperactivity	104	98.1	39	95.1	65	100.0
22. Steals from home, school or elsewhere	Conduct problems	105	99.1	40	97.6	65	100.0
23. Gets on better with adults than with other children	Peer problems	104	98.1	39	95.1	65	100.0
24. Many fears, easily scared	Emotional symptoms	104	98.1	39	95.1	65	100.0
25. Sees tasks through to the end, good attention span	Hyperactivity	77	72.6	38	92.7	39	60.0

Note. The Prosocial scale is excluded from the total difficulties score.

Incomplete data on SDQ individual items caused one participant to have incomplete total scores for the Emotional Symptoms, Conduct Problems, Peer Problems and Total Difficulties scales to be missing and two participants to have a missing total score for the Hyperactivity scale. Although there was a sizeable amount of missing data on individual items of the SDQ, the algorithm used to calculate total scores allows responses on up to two out of the five items for each scale to be missing.

SDQ data were more complete for parents who were not helped by the researcher than those who were helped by the researcher. Of the 41 parents who completed the SDQ without the help of the researcher, 26 (63.4%) had complete data and 15 (36.6%) had incomplete data; 7 (46.7%) of these 15 participants omitted one item, 3 (20%) omitted two items, 4 (26.7%) omitted two items, 4 (26.7%) did not complete three items and 1 (6.7%) did not complete fifteen items. Of the 65 parents who were helped by the researcher to complete the SDQ, 35 (53.8%) had complete data and 30 (46.2%); 25 (83.3%) of the 30 omitted one item, 3 (10%) omitted two items and 2 (6.7%) did not complete three items. The majority (90%) of participants who omitted responding to item 25 (for which the most data was missing) were helped by the researcher to complete the questionnaire.

### Levels of behavioural and emotional problems

Table 4 displays means, standard deviations and range of scores for each of the subscales and total scores on the PEDS and SDQ and the proportion of children with total and subscale scores at or above clinical cut-offs. Overall, children had high scores on total scale and subscale scores on the PEDS and SDQ and the proportion of children meeting clinical cut-offs on both measures was highest for emotional difficulties. Forty nine per cent of children met the cut-off on the PEDS Anxious/Withdrawn subscale, 62.3% on the Fearful subscale and the total for the instrument. Fewer children met the cut-off for behavioural problems as measured with the Acting Out subscale; however, the proportion still amounted to a third of children. Analysis of the frequencies of responses to each of the PEDS trauma-specific items (Table 5) revealed that 50% of children ‘often’ or ‘very often’ brought up the trauma they had experienced in conversation, 54.7% ‘often’ or ‘very often’ appeared fearful of things that were a reminder of the trauma and 42.4% were reported to ‘often’ or ‘very often’ create pictures, stories or games about the traumatic event. In contrast, 81.1% of children were rated as ‘almost never’ or ‘sometimes’ avoiding talking about the traumatic event even when asked. On the SDQ, parents reported that 45.3% of children met the clinical cut-off for emotional symptoms, 37.7% for conduct problems and 38.7% for total difficulties.

**Table 4.** Means and standard deviations of scores on PEDS and SDQ and proportion of participants with scores at or above clinical cut-offs

PEDS	*M*	s.d.	Range	*N* (%) at or above cut-off
Anxious/withdrawn	10.28	3.32	6–22	52 (49.1)
Fearful	10.05	3.82	5–20	66 (62.3)
Acting out	11.87	3.71	6–24	36 (34.0)
Total	32.20	8.53	18–59	66 (62.3)
SDQ	*M*	s.d.	Range	*N* (%) at or above cut-off
Emotional symptoms	4.34	2.28	0–10	48 (45.3)
Conduct problems	3.06	1.99	0–9	40 (37.7)
Hyperactivity	4.85	2.37	0–10	21 (19.8)
Peer problems	2.60	1.88	0–8	28 (26.4)
Prosocial behaviour	6.68	2.69	0–10	20 (18.9)
Total	14.89	6.93	4–32	41 (38.7)

**Table 5.** Frequency of scores on trauma-specific PEDS items

	Almost never		Sometimes		Often		Very often	
PEDS item	*N*	%	*N*	%	*N*	%	*N*	%
18 (Pictures)	29	27.4	32	30.2	26	24.5	19	17.9
19 (Brings up)	29	27.4	24	22.6	24	22.6	29	27.4
20^a^ (Avoids)	69	65.1	17	16.0	8	7.5	11	10.4
21 (Reminders)	30	28.3	18	17.0	34	32.1	24	22.6

Note. 18 = Creates games, stories or pictures about [traumatic event (TE)]; 19 = brings up (TE) in conversation; 20 = avoids talking about (TE) even when asked; 21 = seems fearful of things that are reminders of (TE).

a data missing for one participant.

## Discussion

This study aimed to test the feasibility of recruitment and using two well-validated and brief questionnaires to collect mental health data about refugee children living in and near refugee camps on the Syrian–Turkish border. The results demonstrated that it is feasible to reach and recruit refugees caring for children with research participation information via NGO run primary schools in and near refugee camps using informed consent procedures while at the same time maintaining the safety of researchers and participants. Further, it proved feasible to achieve a good return rate for completed questionnaires for a reasonable sample size of caregivers. This may have been accounted for by the researcher assisting more than half of caregivers with completion of the questionnaires. Although under significant stress, parents appeared able to concentrate and complete measures, and the procedure did not cause apparent distress. The ethics of conducting research in contexts of conflict has been raised (Ford *et al.* 2009). Recently, El-Khani *et al.* (2013) discussed the ethics concerning asking recently displaced families about psychological need, particularly the risk of reactivating distressing accounts of trauma. The authors reported parents were able to talk about parenting experiences, were keen to take part in discussions, stressed the normality of talking about such experiences among themselves and valued the opportunity to share their experiences.

The strong and good working relationship between the research team and Generation Freedom played a key role in making this possible. The safety of the researcher and refugees was a significant concern at all times but was maintained due to expert support from Generation Freedom whose volunteers and employees have extensive experience of working in conflict as well as good local knowledge of the Syrian conflict. Their role was essential in enabling the researcher to cross the Syrian–Turkish border, providing the researcher with access to refugee camps and schools, logistical support to facilitate data collection and ensuring safety of the researcher and refugees at all times. This highlights that while it is possible to conduct research in the midst of a conflict, careful planning and support from expert agencies is vital.

The second aim of the study was to assess the extent to which complete data could be obtained. Firstly, and surprisingly, data for both scales was more complete for participants who were not helped by the researcher compared with those who were helped by the researcher. This was particularly the case for the last item of the SDQ. Participants informed the researcher that they did not wish to respond to particular items or that the items were not applicable to their child. Although the specific reasons for this require further exploration it seems caregivers did not find all items on the SDQ relevant to their situation. Second, while most caregivers completed all items of the PEDS, almost half the caregivers had incomplete data on the SDQ. This may reflect that the PEDS has better specificity for this population and in this context; unlike the SDQ it is designed specifically to measure trauma-related symptoms. It appeared that caregivers found items on the SDQ less applicable in their context. Reliability for the SDQ was poor in this context. This is consistent with studies that have used the SDQ as a secondary outcome measure in evaluations of psychological interventions to reduce post-traumatic stress in Palestinian children (Quota *et al.* 2012; Barron *et al.* 2013). However, the reliability analysis was based solely on Cronbach's alphas and for Total Difficulties and the Hyperactivity scales omitted item 25 due to the large amount of missing data for this item. While the internal consistency for the PEDS was good but not excellent, it offers promise in being a potentially reliable screening tool of child mental health in this context. These findings suggest further research is needed to rigorously test the reliability and validity of these scales in this context. Third, although for individual items there was a significant amount of data missing, the calculation of total scores was not affected for the SDQ as the algorithm for calculating total scores allows up to two items per scale to be missing.

While it was not possible to aim for a large or representative sample in this context, the third aim of the study was to gain insight into the levels of emotional and behavioural difficulties among refugee children living in or near refugee camps on the Turkish–Syrian border. Consistent with previous research on the impact of conflict in the Middle East on internally displaced and refugee children (Dimitry, 2011), results indicated high levels of emotional and behavioural problems measured using both the PEDS and SDQ; elevated mean levels of difficulties for all subscales were found compared with existing normative data apart from SDQ Prosocial Behaviour. Nearly half of children were reported as having clinically significant levels of PEDS rated anxiety and being withdrawn and close to two thirds were fearful. These are higher levels than have been reported using the PEDS in samples of children exposed to natural disaster (Saylor *et al.* 1999). This highlights the need for evidence-based interventions providing psychological support to children and families that are tailored to the nature of the trauma that children have been exposed to. Examining the frequency of scores on trauma-specific PED items, parents reported that the majority of children did not avoid talking about traumatic events. This may have important therapeutic implications on helping children cope effectively with trauma.

A similar percentage of children meeting the cut-off for PEDS anxiety symptoms was found for emotional symptoms reported by parents using the SDQ. The percentage of children (38%) meeting the clinical cut-off for conduct problems rated using the SDQ was comparable with the rate (37%) found in Syrian children using the same measure in the IMC and UNICEF (2013) conducted mental health and psychosocial support (MHPSS) assessment in the Za'atari refugee camp in Jordan. In the same MHPSS assessment, a greater number of children (60% *v.* 45%) had clinically significant scores for SDQ rated emotional symptoms and fewer children (6% *v.* 19%) had prosocial behaviour scores that met the clinical cut-off. Mean levels of emotional symptoms and conduct problems were higher than average levels reported in other studies of refugee children exposed to conflict and resettled in high-income countries (Papageorgiou *et al.* 2000; Fazel & Stein, 2003; Durà-Vilà *et al.* 2012). One might expect to see increased severity of mental health outcomes in children still living in the challenging environment of a refugee camp within close proximity to the conflict compared with resettled children. Prosocial behaviour measured on the SDQ was lower in comparison with norms. Although children may experience a traumatic event, they are still likely to show or increase acts of prosocial behaviour (Thabet *et al.* 2006; Betancourt & Khan, 2008; Masten, 2011). Camps are also believed to become more community-oriented when families live in such close quarters (Dimitry, 2011).

Some methodological considerations must be considered in interpreting the findings. First, opportunistic rather than random sampling was utilized. This is common in refugee camps where the possibility of using random sampling is limited for practical reasons. The extent to which families who participated are representative of the population from where they were recruited is not known. It is possible that the results may underestimate mental health problems in children residing in or near to camps on the Syrian-Turkish border given that those who have better mental health may be more likely to participate in research than those who are less well.

Second, this was a single informant design. Although parents are typically viewed as a prime source of data about their children's emotional and behavioural status, school-age children and their parents may not agree about posttraumatic event exposure symptoms (Belter *et al.* 1991). Further, parents whose own distress is high and who may be experiencing mental health difficulties tend to rate their children's behaviours higher (Saylor *et al.* 1997). However, some evidence suggests that parents tend to underestimate their children's responses to traumatic events (Almqvist & Broberg, 1997; Awadh *et al.* 1998) or on some measures, in general, parent ratings appear to be less reliable than children's self-reports of their own behaviour (Barron *et al.* 2013). Obtaining additional ratings from teachers or other key people in the child's environment may give a more reliable picture. This raises another feasibility question in this context, whether it is possible to obtain proxy reported data on child behaviour and is a question that we could address in our future studies given that we have demonstrated an effective recruitment route via primary schools for refugee children where access to teachers is possible.

Third, evaluation of the reliability of both questionnaires was limited due to the researcher helping many, but not all participants and participants completing questionnaires in different locations (e.g. schools, their tents). However, this study stresses the complexity of being able to reliably collect data in this challenging and chaotic environment. Reliance on Cronbach's alphas only as the sole index of reliability of the scales is not ideal.

Fourth, there are currently no normative data available for the PEDS or SDQ in Syrian or Middle Eastern populations. Therefore cut-offs were based on those used in the USA for the PEDS and on British norms for the SDQ. More research is required to establish cut-offs specific to the culture and context within which refugee children in the Syrian conflict live.

## Conclusion

This study demonstrated an effective method of accessing and recruiting caregivers into a study from primary schools in and near refugee camps on the Syrian–Turkish border and collecting child mental health data in this context, with support provided by an NGO with expert local knowledge and experience of working in the Syrian conflict and while maintaining researcher and caregiver safety. Findings indicated that the PEDS appeared a more reliable tool than the SDQ for assessing refugee children's mental health difficulties. The high levels of emotional and behavioural problems stresses the immediate need for providing evidence-based psychological support to refugee children and families affected by the Syrian conflict. Further feasibility studies are required to identify effective approaches to improve data completeness in the challenging conditions and rigorously test the reliability and also the validity of the measures used.

## References

[ref1] Abou-Saleh M, Mobayed M. (2013). Mental health in Syria. International Psychiatry 10, 58–60. PMC6735115 31507735

[ref2] Almqvist K, Broberg A (1997). Silence and survival: working with strategies of denial in families of traumatized pre-school children. Journal of Child Psychotherapy 23, 417–435.

[ref3] Alyahri A, Goodman R (2006). Validation of the Arabic Strengths and Difficulties Questionnaire and Development. Eastern Mediterranean Health Journal 12, S138–S146. 17361685

[ref4] Attanayake V, McKay R, Joffres M, Singh S, Burkle F Jr, Mills E (2009). Prevalence of mental disorders among children exposed to war: a systematic review of 7920 children. Medicine, Conflict and Survival 25, 4–19. doi:10.1080/13623690802568913. 19413154

[ref5] Awadh AM, Vance B, El-Beblawi V, Pumariega AJ (1998). Effects of trauma of the Gulf War on Kuwaiti children. Journal of Child and Family Studies 7, 493–498. doi: 10.1023/A:1022962127860.

[ref6] Barron IG, Abdallah G, Smith P (2013). Randomised controlled trial of a CBT trauma recovery program in Palestinian schools. Journal of Loss and Trauma: International Perspectives on Stress & Coping 18, 306–321. doi: 10.1080/15325024.2012.688712.

[ref7] Belter RW, Dunn SE, Jeney P (1991). The psychological impact of Hurricane Hugo on children: a needs assessment. Advances in Behaviour Research and Therapy 13, 155–161.

[ref8] Betancourt TS, Khan KT (2008). The mental health of children affected by armed conflict: protective processes and pathways to resilience. International Review of Psychiatry 20, 317–328. 18569183 10.1080/09540260802090363 PMC2613765

[ref9] Bronstein I, Montgomery P (2011). Psychological distress in refugee children: a systematic review. Clinical Child and Family Psychology Review 14, 44–56. doi: 10.1007/s10567-010-0081-0. 21181268

[ref10] Derulyn I, Broekaert E (2007). Different perspectives on emotional and behavioural problems in unaccompanied refugee children and adolescents. Ethnicity and Health 12, 141–162. doi:10.1080/13557850601002296. 17364899

[ref11] Dimitry L (2011). A systematic review on the mental health of children and adolescents in areas of armed conflict in the Middle East. Child: Care, Health and Development 38, 153–161. doi:10.1111/j.1365-2214.2011.01246.x. 21615769

[ref12] Durà-Vilà G, Klasen H, Makatini Z, Rahimi Z, Hodes M (2012). Mental health problems of young refugees: duration of settlement, risk factors and community-based interventions. Clinical Child Psychology and Psychiatry 18, 604–623. doi:10.1177/1359104512462549. 23104967

[ref13] El-Khani A, Ulph F, Redmond AD, Calam R (2013). Ethical issues in research into conflict and displacement [Letter to the editor]. Lancet 382, 764–765. doi:10.1016/S0140-6736(13)61824-3. 23993182

[ref54] Fazel M, Stein A (2002). The mental health of refugee children. Archives of Disease in Childhood 87, 366–370. doi: 10.1136/adc.87.5.366. 12390902 PMC1763071

[ref14] Fazel M, Stein A (2003). Mental health of refugee children: a comparative study. British Medical Journal 327, 134. 12869455 10.1136/bmj.327.7407.134 PMC165700

[ref15] Fazel M, Wheeler J, Danesh J (2005). Prevalence of serious mental disorder in 7000 refugees resettled in western countries: a systematic review. Lancet 365, 1309–1314. 15823380 10.1016/S0140-6736(05)61027-6

[ref16] Ford N, Mills EJ, Zachariah R, Upshur R (2009). Ethics of conducting research in conflict settings. Conflict and Health 3, 7. doi: 10.1186/1752-1505-3-7. 19591691 PMC2717053

[ref17] Frederick C (1985a). Children traumatized by catastrophic situations In Post-Traumatic Stress Disorder in Children (ed. S. Eth and R.S. Pynoos), pp. 73–99. American Psychiatric Press: Washington, DC.

[ref18] Goodman R (1997). The Strengths and Difficulties Questionnaire: a research note. Journal of Child Psychology and Psychiatry 38, 581–586. doi:10.1111/j.1469-7610.1997.tb01545.x. 9255702

[ref19] Goodman R (2001). Psychometric properties of the Strengths and Difficulties Questionnaire. Journal of the American Academy of Child and Adolescent Psychiatry 40, 1337–1345. doi:10.1097/00004583-200111000-00015. 11699809

[ref20] Goodman R, Renfrew D, Mullick M (2000). Predicting type of psychiatric disorder from Strengths and Difficulties Questionnaire (SDQ) scores in child mental health clinics in London and Dhaka. European Child and Adolescent Psychiatry 9, 129–134. doi:10.1007/s007870050008. 10926063

[ref21] IBM Corp (2013). IBM SPSS Statistics for Windows, Version 22.0. IBM Corp: Armonk, NY.

[ref22] International Medial Corps (IMC) & and United Nations Children's Fund (UNICEF) (2013). Mental health/psychosocial and child protection assessment for Syrian refugee adolescents in Za'atari Refugee Camp Jordan. Accessed 25 October 2014 Available from http://reliefweb.int/sites/reliefweb.int/files/resources/IMC%20MHPSS%20and%20CP%20Assessment%20Zaatari%20July%202013%20final%20%281%29.pdf.

[ref23] Khamis V (2005). Post-traumatic stress disorder among school age Palestinian children. Child Abuse & Neglect 29, 81–95. 15664427 10.1016/j.chiabu.2004.06.013

[ref24] Lustig SI, Kia-Keating M, Knight WG, Geltman P, Ellis H, Kinzie H, Keane T, Saxe GN (2004). Review of child and adolescent refugee mental health. Journal of the American Academy of Child and Adolescent Psychiatry 43, 24–36. doi: 10.1097/00004583-200401000-00012. 14691358

[ref25] Masten AS (2011). Resilience in children threatened by extreme adversity: frameworks for research, practice, and translational synergy. Developmental Psychopathology 23, 493–506. doi: 10.1017/S0954579411000198. 23786691

[ref26] Mobayed M (2014). Psychological consequences of the Syrian conflict on Syrian refugees. Paper presented at the Third International Conference of the Jordanian Association of Psychiatrists, Amman, Jordan.

[ref27] Mollica RF, Cardozo BL, Osofsky HJ, Raphael B, Ager A, Salama P (2004). Mental health in complex emergencies. Lancet 364, 2058–2067. 15582064 10.1016/S0140-6736(04)17519-3

[ref28] Mullick MSI, Goodman R (2001). Questionnaire screening for mental health problems in Bangladeshi children: a preliminary study. Social Psychiatry and Psychiatric Epidemiology 36, 94–99. doi:10.1007/s001270050295. 11355451

[ref29] Nielsen SS, Norredam M, Christianse KL, Obel C, Hilden J, Krasnik A (2008). Mental health among children seeking asylum in Denmark – the effect of length of stay and number of relocations: a cross-sectional study. BMC Public Health 8, 293. doi: 10.1186/1471-2458-8-293. 18713455 PMC2535781

[ref30] Özer B, Sirin S, Oppedal B (2013). Bahçeşehir study of Syrian refugee children in Turkey. Bahçeşehir University. Accessed 12 September 2014 Available from http://www.fhi.no/dokumenter/4a7c5c4de3.pdf.

[ref31] Panter-Brick C, Eggerman M, Gonzalez V, Safdar S (2009). Violence, suffering, and mental health in Afghanistan: a school-based survey. Lancet 374, 807–816. doi: 10.1016/S0140-6736(09)61080-1. 19699514 PMC2748901

[ref32] Papageorgiou V, Frangou-Garunovic A, Iordanidou R, Yule W, Smith P, & Vostanis P (2000). War trauma and psychopathology in Bosnian refugee children. European Child and Adolescent Psychiatry 9, 84–90. doi: 10.1007/s007870050002. 10926057

[ref33] Patel V, Flisher AJ, Hetrick S, McGorry P (2007). Mental health of young people: a global public-health challenge. Lancet 369, 1302–1313. doi: 10.1016/S0140-6736(07)60368-7. 17434406

[ref34] Patel V, Flisher AJ, Nikapota A, Malhotra S (2008). Promoting child and adolescent mental health in low and middle income countries. Journal of Child Psychology and Psychiatry 49, 313–334. doi:10.1111/j.1469-7610.2007.01824.x. 18093112

[ref35] Quosh C, Eloul L, Ajlani R (2013). Mental health of refugees and displaced persons in Syria and surrounding countries: a systematic review. Intervention 11, 276–294.

[ref36] Quota A, Punamaki R, Miller T, El-Sarraj E (2008). Does war beget child aggression? Military violence, gender, age, and aggressive behavior in two Palestinian samples. Aggressive Behavior 34, 231–244. 17985361 10.1002/ab.20236

[ref37] Quota S, Punamäki RL, El Sarraj E (2003). Prevalence and determinants of PTSD among Palestinian children exposed to military violence. European Child and Adolescent Psychiatry 12, 265–272. doi: 10.1007/s00787-003-0328-0. 14689258

[ref38] Quota SR, Palosaari E, Diab M, Punamaki RL (2012). Intervention effectiveness among war-affected children: a cluster randomised controlled trial on improving mental health. Journal of Traumatic Stress 25, 288–298. 22648703 10.1002/jts.21707

[ref39] Samad L, Hollis C, Prince M, Goodman R (2005). Child and adolescent psychopathology in a developing country: testing the validity of the strengths and difficulties questionnaire (Urdu version). International Journal of Methods in Psychiatric Research 14, 158–166. 16389892 10.1002/mpr.3 PMC6878532

[ref40] Sanders MR, Markie-Dadds C, Turner KMT (2000). Practitioner's Manual for Standard Triple P. Families International Publishing: Brisbane, QLD, Australia.

[ref41] Saylor CF, Belter R, Stokes SJ (1997). Children and families coping with disasters. Stress and coping in children In Handbook of Children's Coping: Linking Theory and Intervention (ed. S.A. Wolchik and I.N. Sandler), pp. 361–386. Plenum Publishing Company: New York.

[ref42] Saylor CF, Swenson CC, Reynolds SS, Taylor M (1999). The pediatric emotional distress scale: a brief screening measure for young children exposed to traumatic events. Journal of Clinical Child Psychology 28, 70–81. doi: 10.1207/s15374424jccp2801_6. 10070608

[ref43] Stokes S, Saylor CF, Swenson CC, Daugherty T (1995). Comparison of children's behaviors following three types of stressors. Child Psychiatry and Human Development 26, 113–123. doi: 10.1007/BF02353235. 8565647

[ref44] Syed EU, Hussein SA, Mahmud S (2007). Screening for emotional and behavioural problems amongst 5–11-year-old school children in Karachi, Pakistan. Social Psychiatry and Psychiatric Epidemiology 42, 421–427. doi: 10.1007/s00127-007-0188-x. 17450455

[ref45] Thabet AA, Stretch D, Vostanis P (2000). Child mental health problems in Arab children: application of the Strengths and Difficulties Questionnaire. International Journal of Social Psychiatry 46, 266–280. doi: 10.1177/002076400004600404. 11201348

[ref47] Thabet AAM, Karim K, Vostanis P (2006). Trauma exposure in pre-school children in a war zone. British Journal of Psychiatry 188, 154–158. doi: 10.1192/bjp.188.2.154. 16449703

[ref48] Tol WA, Barbui C, Galappatti A, Silove D, Betancourt TS, Souza R, Golaz A, van Ommeren M (2011b). Mental health and psychosocial support in humanitarian settings: linking practice and research. Lancet 378, 1581–1591. doi:10.1016/S0140-6736(11)61094-5. 22008428 PMC3985411

[ref49] Tol WA, van Ommeren M (2012). Evidence-based mental health and psychosocial support in humanitarian settings: gaps and opportunities. Evidence Based Mental Health 15, 25–26. doi:10.1136/ebmental-2012-100644. 22450116

[ref50] United Nations Children's Fund (UNICEF) (2005). UNICEF Annual Report 2004. UNICEF: New York.

[ref51] United Nations Children's Fund (UNICEF) (2013). Syria's Children: A lost Generation? Crises Report March 2011–March 2013. UNICEF: NY. Accessed 14 September Available from: http://www.unicef.org/files/Syria_2yr_Report.pdf

[ref52] United Nations High Commission for Refugees (UNHCR) (2015). 2015 UNHCR country operations profile – Syrian Arab Republic. Accessed 15 February 2015 Available from http://www.unhcr.org/pages/49e486a76.html

[ref53] Vaage AB, Tingvold L, Hauff E, Van Ta T, Wentzel-Larsen T, Clench-Aas J, Thomsen PH (2009). Better mental health in children of Vietnamese refugees compared with their Norwegian peers – a matter of cultural difference? Child and Adolescent Psychiatry and Mental Health 3, 34. doi: 10.1186/1753-2000-3-34. 19845965 PMC2770448

